# Difference between pre-operative and cardiopulmonary bypass mean arterial pressure is independently associated with early cardiac surgery-associated acute kidney injury

**DOI:** 10.1186/1749-8090-5-71

**Published:** 2010-09-08

**Authors:** Hussein D Kanji, Costas J Schulze, Marilou Hervas-Malo, Peter Wang, David B Ross, Mohamad Zibdawi, Sean M Bagshaw

**Affiliations:** 1 Department of Surgery, Faculty of Medicine and Dentistry, University of Alberta, Edmonton, Canada; 2 Mazankowski Alberta Heart Institute, University of Alberta, Edmonton, Canada; 3 Epidemiology Coordinating and Research Centre (EPICORE), University of Alberta, Edmonton, Canada; 4 Division of Critical Care Medicine, Faculty of Medicine and Dentistry, University of Alberta, Edmonton, Canada

## Abstract

**Background:**

Cardiac surgery-associated acute kidney injury (CSA-AKI) contributes to increased morbidity and mortality. However, its pathophysiology remains incompletely understood. We hypothesized that intra-operative mean arterial pressure (MAP) relative to pre-operative MAP would be an important predisposing factor for CSA-AKI.

**Methods:**

We performed a prospective observational study of 157 consecutive high-risk patients undergoing cardiac surgery with cardiopulmonary bypass (CPB). The primary exposure was delta MAP, defined as the pre-operative MAP minus average MAP during CPB. Secondary exposure was CPB flow. The primary outcome was early CSA-AKI, defined by a minimum RIFLE class - RISK. Univariate and multivariate logistic regression were performed to explore for association between delta MAP and CSA-AKI.

**Results:**

Mean (± SD) age was 65.9 ± 14.7 years, 70.1% were male, 47.8% had isolated coronary bypass graft (CABG) surgery, 24.2% had isolated valve surgery and 16.6% had combined procedures. Mean (± SD) pre-operative, intra-operative and delta MAP were 86.6 ± 13.2, 57.4 ± 5.0 and 29.4 ± 13.5 mmHg, respectively. Sixty-five patients (41%) developed CSA-AKI within in the first 24 hours post surgery. By multivariate logistic regression, a delta MAP≥26 mmHg (odds ratio [OR], 2.8; 95%CI, 1.3-6.1, p = 0.009) and CPB flow rate ≥54 mL/kg/min (OR, 0.2, 0.1-0.5, p < 0.001) were independently associated with CSA-AKI. Additional variables associated with CSA-AKI included use of a side-biting aortic clamp (OR, 3.0; 1.3-7.1, p = 0.012), and body mass index ≥25 (OR, 4.2; 1.6-11.2, p = 0.004).

**Conclusion:**

A large delta MAP and lower CPB flow during cardiac surgery are independently associated with early post-operative CSA-AKI in high-risk patients. Delta MAP represents a potentially modifiable intra-operative factor for development of CSA-AKI that necessitates further inquiry.

## Introduction

Acute kidney injury (AKI) following cardiac surgery with cardiopulmonary bypass (CPB) can be a devastating complication associated with high morbidity, mortality and resource utilization [1,2]. The incidence of cardiac surgery-associated AKI (CSA-AKI) has ranged between 5-30% [3,4]. This variability is largely attributable to the numerous definitions applied in prior studies and assessment of inconsistent at-risk patient populations. Severe AKI prompting initiation of renal replacement therapy (RRT) after cardiac surgery is uncommon, however, has been associated with a 7.9 fold increased risk of death [5]. However, even relatively mild rises in serum creatinine in the immediate post-operative period have been associated with reduced survival [6].

Despite the deleterious impact of CSA-AKI on outcome, its pathophysiology remains incompletely understood. The extracorporeal circuit and CPB have been implicated as key contributing factors [7,8]. In particular, pre-existing chronic kidney disease (CKD), prolonged aortic cross clamp and CBP duration have been found to predict CSA-AKI [9,10]. In general, however, there is a paucity of data that has focused on the association between specific intra-operative CPB parameters and risk of CSA-AKI [11].

Accordingly, we performed a prospective observational study of patients undergoing cardiac surgery with CPB at high-risk for CSA-AKI. Our objective was to evaluate for associations between intra-operative CPB parameters and early post-operative CSA-AKI. Specifically, we examined the effect of: 1) delta mean arterial pressure (MAP); and 2) CPB flow on the risk for early post-operative CSA-AKI.

## Methods

### Study Design

This was a prospective observational cohort study. Consecutive patients undergoing cardiac surgery with CPB at the Mazankowski Alberta Heart Institute, University of Alberta Hospital in Edmonton, Canada between July 1, 2008 and October 31, 2008 were screened for enrollment. The cardiac surgery program has eight surgeons who perform approximately 1400 open heart cases with CPB per year. The Health Research Ethics Board at the University of Alberta approved the protocol prior to commencement.

### Study Population

**Figure 1:**
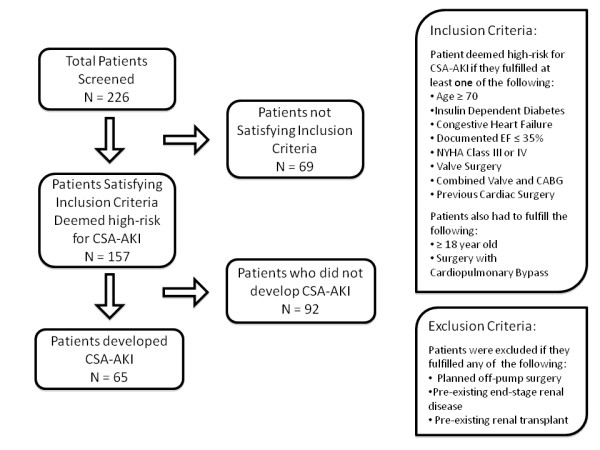
**Patient Flow Chart**.

Patients with features putting them at risk for CSA-AKI were recruited for this study. For this study, patients deemed "high-risk" were adopted from Thakar et al [12-14] and included patients who had at least one of the following: age ≥70 years; insulin-dependent diabetes mellitus (DM); congestive heart failure or documented LVEF <35%; New York Heart Association (NYHA) symptom severity class 3 or 4; pre-operative serum creatinine ≥106 μmol/L; valve surgery only; valve surgery + CABG or complex surgery; and/or previous cardiac surgery. Inclusion criteria were adult patients (age ≥18 years) undergoing cardiac surgery with CPB and presence of at least 1 high-risk criterion. Exclusion criteria included: planned off-pump cardiac surgery; cardiac or lung transplantation; isolated ventricular device insertion; and end-stage kidney disease (CKD class V) or prior kidney transplantation.(Figure 1)

### Study Definitions

Acute kidney injury (AKI) was defined using the RIFLE classification scheme where the three strata of injury were defined as: i) RISK - serum creatinine increase ×1.5 baseline or urine output <0.5 ml/kg/hour × 6 hours, ii) INJURY - serum creatinine increase ×2.0 or urine output <0.5 ml/kg/hour × 12 hours, and iii) FAILURE - serum creatinine ×3.0 or urine output <0.3 ml/kg/hour × 24 hours or anuria for 12 hours [15]. We ascertained for AKI within the first 24 post-operative hours after cardiac surgery. The rationale for this "early" definition was to capture AKI most likely attributable to intra-operative factors such as CPB, rather than factors in the post-operative period. Delta MAP was defined as baseline MAP (acquired from three independent pre-operative blood pressure readings) minus the average MAP on CPB (calculated as the average of MAP readings at 15 minute intervals during CPB).

### Study Protocol

For those patients enrolled, detailed data collection was performed. All data were extracted using standardized case-report forms and entered into a central Access 2003 database (Microsoft Corp, Richmond, USA). Data extracted included: demographics (e.g. age, sex, pre-morbid illness, pre-operative medications), pre-operative kidney function, surgical details (e.g. coronary bypass, value replacement, technique, cross-clamp time), intra-operative parameters (e.g. mean perfusion pressure, flow, concomitant ultrafiltration, temperature, hematocrit, transfusions, use of vasoactive medication, use of anti-fibrinolytics) and post-operative details (e.g. clinical, physiologic and laboratory data). Data were also ascertained on clinical outcomes including: occurrence of AKI, receipt of RRT, duration of mechanical ventilation, lengths of stay and hospital mortality. Postoperative data was collected for 5 days. Pre-operative MAP was calculated as an average of three distinct measurements of blood pressure separated by greater than 24 hours between readings. Two of the measurements were conducted preoperatively using an automated blood-pressure cuff (pre-admission clinic and on admission to the hospital), the third was extracted from anesthesiologist's record prior to administration of anesthesia from the radial arterial line.

### Operation and CPB

All surgeries were performed through a midline sternotomy with the use of CPB. CPB was instituted using standard techniques with cannulation of the right atrium with a 42F cavoatrial venous cannula and the ascending aorta with a 20 or 22F aortic cannula. In the case of mitral valve surgery a bicaval cannulation technique with a 30F SVC- and 34F IVC-cannula was employed for venous drainage. A phosphorylcholin coated membrane oxygenator (Dideco 903 Avant™) and roller pump (Stöckert S-3 or S-5, Stöckert Instrument GmbH, Munich, Germany) was used in all patients. The phosphorylcholin coated (PHISIO™, Dideco, Mirandola, Italy) circuit was primed with Plasma-Lyte^® ^500 ml, Pentaspan^® ^500 ml, Mannitol 25 g and 10000 units of unfractionated heparin (UH). Permissive hypothermia was allowed, temperature was measured with a rectal probe and maintained at >33°C.

UH (400 units/kg) was given prior to cannulation. Activated clotting time was maintained at ≥480 seconds during the procedure. Nonpulsatile pump flow rates were kept at 2.4 L/min/m^2 ^and the MAP was adjusted to keep the surgical field bloodless and to avoid severe hypotension <50 mmHg. In general the targeted MAP was 60 mmHg. To maintain the filling volume of the extracorporeal circuit, colloids (Pentaspan^®^) and Ringer's Lactate solution were added. When the hemoglobin was less than 70 g/L, packed red blood cells were transfused. Blood cardioplegia with modified Buckberg solution at a ratio of 4:1 with high potassium (20 mmol/L) at induction, and at a ratio of 16:1 with low potassium (8 mmol/L) for maintenance was used for myocardial protection. Cardioplegic solution was delivered in an antegrade fashion via the aortic root or by direct cannulation of the coronary ostia or in a retrograde fashion via the coronary sinus. Heparin was reversed with protamine following decannulation.

Patients were transferred to the cardiovascular surgical intensive care unit post-operatively. All fluid, inotropes, hemodynamics and lab values including creatinine were recorded for 5 days post-operatively. Post-operative patient management included radial arterial pressure monitoring and in some cases thermodilution pulmonary artery catheters (Baxter Healthcare Corp, Santa Ana, USA) to measure cardiac index. Patients were extubated from mechanical ventilation at the discretion of the intensivist according to standard weaning protocols. All procedure specific data is reported on Table 1.

**Table 1:** Baseline demographic and pre-operative characteristics stratified by post-operative CSA-AKI.

Characteristic	No AKI (n = 92)	AKI (n = 65)	p-value
Age (years) (mean[± SD])	64.7 ± 15.8	67.5 ± 13	0.33
Male Sex (%)	64 (69.6)	46 (70.8)	0.87
BMI (kg/m^2^) (mean[± SD])	26.3 ± 4.1	31.5 ± 7.1	< 0.0001
CAD (%)	42 (45.7)	38 (58.5)	0.11
Angina (%)	8 (8.7)	5 (7.7)	0.83
Previous MI	35 (38)	31 (47.7)	0.23
Previous Revascularization (%)	9 (9.8)	4 (6.2)	0.42
Valve disease (%)	52 (56.5)	27 (41.5)	0.06
HTN (%)	51 (55.4)	44 (67.7)	0.12
DM - Insulin-Dependent (%)	8 (8.7)	14 (21.5)	0.02
DM - Non Insulin Dependent (%)	23 (25)	24 (36.9)	0.12
Dyslipidemia (%)	57 (62)	48 (73)	0.12
PVD (%)	9 (9.8)	11 (16.9)	0.19
CVD (%)	9 (9.8)	5 (7.7)	0.65
Creatinine (μmol/L) (mean[± SD])	102.1 ± 29.3	100.3 ± 24.1	0.98
Chronic Kidney Disease (%)	12 (13)	9 (13.8)	0.88
Pre-op SBP (mm Hg) (mean [± SD])	123.6 ± 21.1	129.5 ± 20.9	0.07
Pre-op DBP (mm Hg) (mean [± SD])	66.5 ± 13.3	67.4 ± 13.3	0.66
Pre-op MAP (mm Hg) (mean [± SD])	85.5 ± 13.2	88.1 ± 13.2	0.22
EF (%)(mean [± SD])	48.4 ± 13.2	47.5 ± 13.6	0.55
ASA (%)	76 (82.6)	54 (83.1)	0.90
Clopidogrel (%)	12 (13.0)	9 (13.8)	0.88
Beta-Blocker (%)	62 (67.5)	45 (69.2)	0.81
CCB (%)	17 (18.5)	11 (16.9)	0.08
ACE inhibitor (%)	56 (60.9)	31 (47.7)	0.10
ARB (%)	6 (6.5)	3 (4.6)	0.62
Statin (%)	60 (65.2)	46 (70.3)	0.46
Loop Diuretic (%)	34 (37)	28 (43.1)	0.44
Thiazide (%)	35 (38)	31 (47.7)	0.23
Spironolactone (%)	2 (2.2)	4 (6.2)	0.23

Abbreviations: DM = diabetes mellitus; BMI = body mass index; AKI = acute kidney injury; CAD = Coronary artery disease, HTN = Hypertension, PVD = peripheral vascular disease, CVD = cerebro-vascular disease, EF = ejection fraction, ASA = acetylsalicylic acid, CCB = Calcium channel blocker, ACE = Angiotensin converting enzyme, ARB = Angiotensin receptor blocker

### Statistical Analysis

The primary outcome was incidence of CSA-AKI, defined by fulfillment of a minimum RIFLE class - RISK. Patient demographic, clinical, physiologic and laboratory data for the pre- and intra-operative periods were summarized as means (± SD) or medians (intraquartile ranges [IQR]), and numbers or proportions and compared using Wilcoxon rank tests, t-tests and chi-square tests, as appropriate. In the event of missing data values, data were not replaced or estimated. We evaluated delta MAP and CPB flow both as continuous variables and dichotomized using an outcome-oriented cut-off method. Delta MAP, selected clinical factors (i.e. age, sex) and additional factors found significant by univariate analysis (p < 0.2) were candidates for multivariable logistic regression. The model was evaluated for colinearity. The final parsimonious model was based on clinical and statistically significant variables. Model fit was assessed by the Hosmer and Lemeshow goodness-of-fit test (c-statistic). Data are presented as odds ratios (OR) with 95% confidence intervals (CI). P-value < 0.05 was considered statistically significant for all comparisons.

## Results

Of the 226 patients screened, 157 fulfilled eligibility criteria (Figure 1). Sixty-five patients (41%) developed CSA-AKI within in the first 24 hours post-surgery. Table 1 displays the details of patient baseline demographics and clinical characteristics prior to CPB. Those patients developing CSA-AKI were more likely to have insulin-dependent DM (21.5% vs. 8.7%, p = 0.02) and a higher mean body mass index (BMI) than in the non-AKI group (31.5 vs. 26.3, p < 0.0001). There was no significant difference in preoperative medications, including operative day administration, between the two groups.

### Delta MAP, CPB Flow and CSA-AKI

A summary of intra-operative parameters stratified by AKI are presented in Table 2. No patient received aprotinin. By univariate analysis, average delta MAP was not significantly different between AKI and non-AKI groups (28.0 ± 13.2 mmHg vs. 31.3 ± 13.8 mmHg, p = 0.10). However, in multivariate analysis, expressing delta MAP as a continuous variable, every one percent increase in delta MAP, significantly increased the odds of AKI increased by 3% after adjustment of other covariates (OR 1.03, 1.0-1.07, p = 0.05, C-statistic = 0.783). Moreover, for patients with a delta MAP ≥26 mmHg, there was a 2.1-fold (95% CI, 1.1-4.2, p = 0.024) increased odds for CSA-AKI (Table 3). A delta MAP ≥26 mmHg was found to be independently associated with CSA-AKI in multi-variable analysis (OR 2.8; 95% CI, 1.3-6.1, p = 0.009, Table 4).

**Table 2:** Summary of intra-operative variables stratified by post-operative CSA-AKI.

Variable	No AKI (n = 92)	AKI (n = 65)	p-value
Valve only surgery (%)	26 (28.3)	12 (18.5)	0.16
Combined (valve + CABG) (%)	43 (46.7)	21 (32.3)	0.07
Re-operation (%)	8 (8.7)	6 (9.2)	0.91
# Grafts (mean [± SD])	3.4 ± 1.1	3.5 ± 1.1	0.77
Duration of CPB (min, mean [± SD])	126.6 ± 52	127.2 ± 63.2	0.69
Duration of cross clamp (min, mean [± SD])	90.9 ± 46.9	88.7 ± 57.1	0.42
Average CPB MAP (mmHg, mean [± SD])	57.8 ± 5.1	56.9 ± 4.9	0.25
Minutes <MAP 60 mmHg (median [± IQR])	59 ± 65	56 ± 45	0.49
Minutes <MAP 50 mmHg (median [± IQR])	2.5 ± 10	5.0 ± 15	0.35
Delta MAP (mmHg, mean [± SD])	28.0 ± 13.2	31.3 ± 13.8	0.10
PRBC transfusions (units, mean [± SD])	1.8 ± 1.5	2.4 ± 2.3	0.27
Patients transfused with PRBC (%)	23 (25)	19 (29.2)	0.56
Insulin dose (Units, mean [± SD])	3.3 ± 1.3	3.6 ± 3.1	0.72
Furosemide dose (mg, n = 9, n = 7, mean [± SD])	22.8 ± 10.3	27.1 ± 12.5	0.50
Ultrafiltration (mL, n = 34, n = 25, mean [± SD])	1440 ± 1049	1470 ± 1344	0.98
Received tranexamic acid (%)	83 (90.2)	58 (89.2)	0.84
Received aprotinin (%)	0 (0)	0 (0)	NS
Use of side-biting clamp (%)	16 (17.4)	21 (32.2)	0.03
Average flow (mL/kg/min, mean[± SD])	60.9 ± 7.1	55.5 ± 8.4	0.001
Average temperature (°C, mean [± SD])	35.3 ± 1.4	35.5 ± 1.1	0.75

Abbreviations: AKI = acute kidney injury; CABG = coronary artery bypass graft, CPB = cardiopulmonary bypass; MAP = mean arterial pressure; PRBC = packed red blood cell

**Table 3:** Univariate Factors associated with early CSA-AKI.

Predictor	Odds Ratio	95% CI	P-value
Male Sex	1.06	0.5-2.1	0.87
Age (per year)	1.01	0.99-1.04	0.25
Age ≥ 75 years (present)	1.7	0.8-3.5	0.15
BMI (kg/m^2^)(per 1 point)	1.2	0.8-3.5	< 0.0001
BMI ≥25 kg/m^2 ^(present)	4.4	1.9-10.2	0.0007
Valve disease (present)	0.55	0.3-1.0	0.06
DM (present)	2.2	1.1-4.2	0.025
PVD (present)	1.9	0.9-3.3	0.19
HTN (present)	1.7	0.9-3.3	0.12
Delta MAP (per 1 mmHg)	1.02	0.99-1.04	0.14
Delta MAP ≥26 mmHg (present)	2.1	1.1-4.2	0.024
Flow ≥54 per mL/kg/min (present)	0.2	0.1-0.5	0.0002
pH	1.4	0.8-2.7	0.26
Pre-operative ACE inhibitor (present)	0.6	0.3-1.1	0.1
Valve Surgery (present)	0.5	0.3-1	0.07
Peak CPB-MAP	0.5	0.2-0.97	0.04
Pre-operative Systolic BP (≥111 mmHg)	2.1	0.99-4.6	0.05
Duration of CPB MAP ≤60 (per 1 min)	1.99	0.9-4.4	0.89

Abbreviations: BMI = Body Mass Index; DM = Diabetes Mellitus PVD = Peripheral Vascular Disease; HTN = Hypertension; MAP = Mean Arterial Pressure; ACE = Angiotensin Converting Enzyme; CPB=Cardiopulmonary Bypass' MAP = mean arterial pressure

**Table 4:** Multi-variable adjusted logistic regression model^¶ ^of association between delta MAP and CSA-AKI.

Parameter	Odds Ratio	95% CI	P-value
Male sex	0.7	0.3-1.7	0.49
Age ≥75 years (present)	2.1	0.9-4.9	0.08
BMI ≥25 kg/m^2 ^(present)	4.2	1.6-11.2	0.0039
Delta MAP ≥26 mmHg (present)	2.8	1.3-6.1	0.009
Flow ≥54 per mL/kg/min (present)	0.3	0.1-0.7	0.004
Side-biting clamp (present)	3.0	1.3-7.1	0.012

Abbreviations: BMI = Body Mass Index; MAP = mean arterial pressure; CPB = cardiopulmonary bypass

Model characteristics: C-statistic = 0.788

A higher CPB flow rate was associated with lower odds of CSA-AKI. Univariate analysis demonstrated that CPB flow in the non-AKI group was significantly higher (60.9 ml/kg/min vs. 55.5 ml/kg/min, OR 0.2; 95% CI, 0.1-0.5, p < 0.01) (Tables 2 and 3). By multivariable analysis, an average blood flow on CPB is ≥54 ml/kg/min was associated with a significantly lower odds of CSA-AKI (OR 0.3; 95% CI, 0.1-0.7, p = 0.004, Table 4). In addition, in this model, both a BMI ≥25 kg/m^2 ^and use of an intra-operative side-biting clamp were independently associated with greater odds of CSA-AKI (Table 4). In the second multivariable model with delta MAP as a continuous variable, both BMI as a continuous variable (OR 1.2; 95% CI, 1.1-1.3, p < 0.0001) and use of a side-biting clamp (OR 2.4; 95% CI, 1.04-5.8, p = 0.039) remained independently associated with higher odds of AKI.

No other intra-operative factors were significantly associated with early CSA-AKI. Specifically, no differences were noted by number of coronary bypass grafts, type of surgery preformed, and duration of either aortic cross clamp or CPB.

### Sensitivity Analysis

A sensitivity analysis was conducted using a different validated definition of AKI (creatinine increase of greater than 25% or 44.2 μmol/L). This sensitivity multivariable model, after adjusting for confounders, showed similar independent associations between delta MAP, CPB flow, use of side-biting clamp and elevated BMI and development of post-operative CSA-AKI (Additional file 1).

The peak delta serum creatinine over the first 5 post-operative days was 22.9 μmol/L (+/- 27.2). When stratified by a delta MAP, the peak delta serum creatinine values were 24.9 μmol/L (+/- 26.4) for delta MAP ≥26 mmHg and 20.3 μmol/L (+/- 28.4) delta MAP <26 mmHg.

### Clinical Outcomes and CSA-AKI

Post-operative outcomes, including time on mechanical ventilation, length of ICU stay were similar between those with and without CSA-AKI (Table 5). No patient received acute RRT and all patients survived to hospital discharge.

**Table 5:** Summary of post-operative clinical outcomes.

	No AKI (n = 92)	AKI (n = 65)	p-value
ICU Duration (hours) (median [IQR])	53 (19-87)	65 (30-100)	0.86
Ventilation duration (hours, median [IQR])	15 (8-22)	15 (6-24)	0.48
RRT (%)	0	0	NS
Death (%)	0	0	NS
Creatinine baseline (μmol/L, mean [± SD])	102.1 ± 29.3	100.3 ± 24.1	0.98
Creatinine Day 1 (μmol/L, mean [± SD])	107.6 ± 31.4	114.3 ± 27.1	0.03
Creatinine Day 2 (μmol/L, mean [± SD])	109.4 ± 37.0	121.4 ± 35.5	0.003
Creatinine Day 3 (μmol/L, mean [± SD])	101.4 ± 32.9	116.5 ± 40.4	0.0003
Creatinine Day 4 (μmol/L, mean [± SD])	97.1 ± 32.9	111.8 ± 45.5	0.011
Creatinine Day 5 (μmol/L, mean [± SD])	97.6 ± 29.6	115.3 ± 44.5	0.02
Creatinine peak 5-day difference (μmol/L, mean [± SD])	16.53 ± 17.68	31.69 ± 34.88	0.002
Urine Output 12 hours (ml/kg/hr, mean [± SD])	1.3 ± 0.6	0.8 ± 0.4	0.002
Urine Output 24 hours (ml/kg/hr, mean [± SD])	1.0 ± 0.8	0.5 ± 0.5	< 0.0001
Urine Output 36 hours (ml/kg/hr, mean [± SD])	1.1 ± 0.8	1.0 ± 0.7	0.02
Urine Output 48 hours (ml/kg/hr, mean [± SD])	1.1 ± 0.7	0.8 ± 0.5	0.38
Highest MAP 5 days post op (mean [± SD])	97.1 ± 13.0	95 ± 12.9	0.25
Lowest MAP in 5 days post-op (mean [± SD])	67.6 ± 9.1	64.6 ± 7.3	0.07

Abbreviations: ICU = intensive care unit, RRT = renal replacement therapy

## Discussion

We performed a prospective observational study of 157 cardiac surgery patients receiving cardiopulmonary bypass at elevated risk for CSA-AKI to evaluate the impact of intra-operative variables, specifically delta MAP and CPB flow, on the development of early post-operative CSA-AKI.

We found early post-operative AKI was common, occurring in 41% of patients. While this would appear significantly higher than prior studies, our study was focused on patients at higher risk for CSA-AKI. In two observational studies of CSA-AKI, defined by the RIFLE criteria, the post-operative incidence of CSA-AKI ranged 3.7-9%(16, 17). In addition, we found that a delta MAP ≥26 mmHg was independently associated with development of early CSA-AKI. More specifically, every 1% increase in delta MAP was found to be associated with a 3% higher risk of CSA-AKI. We found that CPB circuit flow <54 mL/kg/min was independently associated with higher risk of early post-operative AKI. We also found that higher BMI (> 25 kg/m^2^) and the intra-operative use of a side-biting aortic clamp were associated with higher risk of early post-operative AKI. While we used the relatively sensitive RIFLE criteria to define AKI, we also found these results were robust when defining CSA-AKI as an increase in creatinine of >25% or >44.2 μmol/L in sensitivity analysis.

By identifying a high-risk cohort we were able to prospectively evaluate important and potentially modifiable peri-operative factors [12]. A recent analysis of the RIFLE criteria was conducted by Kuitunen et al [18], which showed that patients undergoing cardiac surgery fulfilling AKI-R criteria (the definition employed in this study) have an 8% 30-day mortality rate compared to 0.9% in the non-risk population. A similar phenomenon was shown by Dasta et al, using the same AKI definition, who reported that even minor elevations of creatinine in the AKI-R group was associated with 2.2 fold greater mortality, 1.6 fold greater ICU length of stay and 1.6 fold greater post operative costs when compared to controls [17]. In light of this data we have focused not on clinical outcomes, but on the immediate post-operative period, to study the influence of delta MAP and CPB flow as contributing factors to the development of CSA-AKI.

Our study is the first to specifically examine the impact of delta MAP, or patient-specific relative hypotension, on peri-operative risk of CSA-AKI. We demonstrate that in addition to a fractional increase in *delta-MAP*, a drop in MAP ≥26 mmHg from preoperative baseline blood pressure is associated 2.8 times greater risk for the development of CSA-AKI. An absolute prolonged drop in pressure <60 mmHg has previously been identified as risk factor for CSA-AKI [7,19]. Furthermore, poorer neurological outcomes and end-organ perfusion have been associated with CPB pressures <60 mm Hg [20]. The role of relative hypotension during CPB remains debated and data exists to suggest that absolute hypotension while on CPB alone is not associated with the development of CSA-AKI [21]. Despite the ongoing discussion on role of perfusion pressure, there is a convincing data to suggest that increased CPB duration has deleterious effects on kidney function and promotes injury [1,10,22]. Unfortunately the majority of the studies that report on CPB duration did not include CPB hemodynamics, specifically CPB-MAP, in the analysis and none of the studies evaluate the change relative to pre-operative pressures (i.e. delta MAP) [1,22]. We identified only one study that related higher post-operative complications defined as composite outcome of cardiac death, CHF, or rise in SCr >20% with a relative drop in intra-operative blood pressure >20 mmHg or 20% [23]. A recent study by Aronson et al demonstrated that pre-operative hypertension with an increase in pulse pressure is an independent risk factor for AKI [24]. Our study would argue that hypertension might be a surrogate marker for a greater relative drop in CPB MAP, which might contribute to CSA-AKI.

The literature has examples of studies that refute CPB hypotension as an independent risk factor for CSA-AKI, however, these studies are generally small, observational, underpowered, and more importantly, these studies failed to investigate the impact of hypotension as a function of pre-operative baseline MAP [20,25-27]. The notion of *relative *hypotension or delta-MAP induced end-organ injury has been recently shown. Gottesman et al found that patients with greater drops of MAP on CPB relative to pre-operative MAP had poorer neurological outcomes [28]. Furthermore, Lombardi et al demonstrated that lower MAP during CPB was an independent risk factor in the development of CSA-AKI [8]. This study suggested low CPB MAP is a potentially important determinant for CSA-AKI, however, does not correlate duration of hypotension to pre-operative baseline. In addition, the study showed a difference of only 0.5 mmHg between cohorts, which though statistically different, may have limited clinical relevance. Though Lomabrdi et al suggest that hypotension during CPB in general could have deleterious effect on post-operative kidney function, our study has shown that the magnitude of injury may be more a function of the degree of hypotension relative to pre-operative baseline MAP.

Cardiopulmonary pressure and flow are intimately related and both are important to preserve end organ perfusion. Currently, there is controversy regarding the superiority of CPB flow delivered as pulsatile or non-pulsatile [29]. Surprisingly, there is paucity in the literature describing the effect of flow on CSA-AKI. Those studies describing the effect of CPB flow on post-operative complications, by in large, focus on neurological outcomes after cardiac surgery [30,31]. Our study identifies CPB flow as an independent factor associated with increasing the likelihood of post-operative CSA-AKI. We found that higher flow rates may be protective and associated with prevention of CSA-AKI.

Many theories surrounding the initiation of inflammatory pathways to hemodynamics, in particular CPB hypotension and flow have been proposed with little supporting evidence [25,32]. CPB related practices, in particular perfusion pressure and flow, are by in large founded on empirical practices and lack the scientific basis to serve as evidence-based guidelines [11,33]. The literature surrounding pump hemodynamics and effect on physiology and clinical outcomes is surprisingly scarce, in particular relating to CSA-AKI. Our study suggests that there should be a concerted effort in re-evaluating strategies surrounding CPB practices and influence on CSA-AKI. Our findings suggest that maintaining a delta MAP <26 mm Hg may be important during CPB to prevent CSA-AKI. Increasing the perfusion pressure can be accomplished by either elevating systemic vascular resistance pharmacologically (which may theoretically reduce renal perfusion) or by increasing CBP flow. As we found the latter to also be protective against CSA-AKI, we would suggest that this be considered first; however, we also caution that confirmatory studies are needed.

Our study has notable limitations. Firstly, our study is single centered, relatively small and observational in nature making it prone to bias. Not being able to control for interventions may have resulted in patients who were deemed high-risk to be maintained intra-operatively at a higher MAP. Secondly, our study may have been subjected to a selection bias, for example a certain surgeon may be more apt to operate on more complicated and higher risk patients with different intra-operative practices. Thirdly, the small sample and relatively sensitive definition for AKI used in our study, coupled with a short post-operative study period, largely limited our statistical power and precluded us from detecting potentially meaningful differences in clinical outcomes, such as duration of mechanical ventilation, duration of ICU stay and need for RRT. As aforementioned, this was in part intended, in order to isolate as best as possible the impact of intra-operative hemodynamic variables on risk of post-operative CSA-AKI. We attempted to control for available confounders by applying an *a priori *selection criteria and collection of factors that could contribute to CSA-AKI. These factors were included in multivariable analysis. Finally, we recognize for the aforementioned reasons, our single-centre study of patients undergoing cardiac surgery with CPB at higher risk for CSA-AKI has limited overall generalizability, when taken in context to patients at lower risk for CSA-AKI or those receiving cardiac surgery at other institutions or in other jurisdictions.

In summary, despite the above mentioned limitations, our study is the first prospective study to focus on the association between delta MAP and post-operative CSA-AKI. A large delta MAP and lower CPB flow are independently associated with development of early post-operative CSA-AKI in patients with prior high-risk features. These factors are potentially identifiable and modifiable. We contend these factors require further investigation.

## Competing interests

The authors declare that they have no competing interests.

## Authors' contributions

HK developed study protocol, obtained data, analyzed data and wrote manuscript. CS obtained data and provided critical revision of manuscript. PW obtained data. DR and MZ developed the study protocol and provided critical revision of the manuscript. MH analyzed data. SMB conceived the study, developed study protocol, analyzed data and provided critical revision of the manuscript. All authors read and approved the final manuscript.

## Supplementary Material

Additional file 1 **Sensitivity multi-variable analysis exploring the association of delta MAP and CPB flow rate on post-operative CSA-AKI using an alternative definition for CSA-AKI**. Click here for file

## References

[B1] Conlon PJ Stafford-Smith M White WD Newman MF King S Winn MP Acute renal failure following cardiac surgery Nephrol Dial Transplant 1999 14 5 1158 62 10.1093/ndt/14.5.1158 10344355

[B2] Liangos O Wald R O'Bell JW Price L Pereira BJ Jaber BL Epidemiology and outcomes of acute renal failure in hospitalized patients: a national survey Clin J Am Soc Nephrol 2006 1 1 43 51 10.2215/CJN.00220605 17699189

[B3] Abu-Omar Y Ratnatunga C Cardiopulmonary bypass and renal injury Perfusion 2006 21 4 209 13 10.1191/0267659106pf870oa 16939114

[B4] Bove T Calabro MG Landoni G Aletti G Marino G Crescenzi G The incidence and risk of acute renal failure after cardiac surgery J Cardiothorac Vasc Anesth 2004 18 4 442 5 10.1053/j.jvca.2004.05.021 15365924

[B5] Chertow GM Levy EM Hammermeister KE Grover F Daley J Independent association between acute renal failure and mortality following cardiac surgery Am J Med 1998 104 4 343 8 10.1016/S0002-9343(98)00058-8 9576407

[B6] Zakeri R Freemantle N Barnett V Lipkin GW Bonser RS Graham TR Relation between mild renal dysfunction and outcomes after coronary artery bypass grafting Circulation 2005 112 9 Suppl I270 5 16159830 10.1161/CIRCULATIONAHA.104.522623

[B7] Fischer UM Weissenberger WK Warters RD Geissler HJ Allen SJ Mehlhorn U Impact of cardiopulmonary bypass management on postcardiac surgery renal function Perfusion 2002 17 6 401 6 10.1191/0267659102pf610oa 12470028

[B8] Lombardi R Ferreiro A Risk factors profile for acute kidney injury after cardiac surgery is different according to the level of baseline renal function Ren Fail 2008 30 2 155 60 10.1080/08860220701808129 18300114

[B9] Del Duca D Iqbal S Rahme E Goldberg P de Varennes B Renal failure after cardiac surgery: timing of cardiac catheterization and other perioperative risk factors Ann Thorac Surg 2007 84 4 1264 71 10.1016/j.athoracsur.2007.05.016 17888981

[B10] Palomba H de Castro I Neto AL Lage S Yu L Acute kidney injury prediction following elective cardiac surgery: AKICS Score Kidney Int 2007 72 5 624 31 10.1038/sj.ki.5002419 17622275

[B11] Bartels C Gerdes A Babin-Ebell J Beyersdorf F Boeken U Doenst T Cardiopulmonary bypass: Evidence or experience based? J Thorac Cardiovasc Surg 2002 124 1 20 7 10.1067/mtc.2002.121506 12091804

[B12] Thakar CV Arrigain S Worley S Yared JP Paganini EP A clinical score to predict acute renal failure after cardiac surgery J Am Soc Nephrol 2005 16 1 162 8 10.1681/ASN.2004040331 15563569

[B13] Haase M Haase-Fielitz A Bellomo R Devarajan P Story D Matalanis G Sodium bicarbonate to prevent increases in serum creatinine after cardiac surgery: a pilot double-blind, randomized controlled trial Crit Care Med 2009 37 1 39 47 10.1097/CCM.0b013e318193216f 19112278

[B14] Burns KE Chu MW Novick RJ Fox SA Gallo K Martin CM Perioperative N-acetylcysteine to prevent renal dysfunction in high-risk patients undergoing cabg surgery: a randomized controlled trial JAMA 2005 294 3 342 50 10.1001/jama.294.3.342 16030279

[B15] Bellomo R Ronco C Kellum JA Mehta RL Palevsky P Acute renal failure - definition, outcome measures, animal models, fluid therapy and information technology needs: the Second International Consensus Conference of the Acute Dialysis Quality Initiative (ADQI) Group Crit Care 2004 8 4 R204 12 10.1186/cc2872 15312219 PMC522841

[B16] Heringlake M Knappe M Vargas Hein O Lufft H Kindgen-Milles D Bottiger BW Renal dysfunction according to the ADQI-RIFLE system and clinical practice patterns after cardiac surgery in Germany Minerva Anestesiol 2006 72 7-8 645 54 16865083

[B17] Dasta JF Kane-Gill SL Durtschi AJ Pathak DS Kellum JA Costs and outcomes of acute kidney injury (AKI) following cardiac surgery Nephrol Dial Transplant 2008 23 6 1970 4 10.1093/ndt/gfm908 18178605

[B18] Kuitunen A Vento A Suojaranta-Ylinen R Pettila V Acute renal failure after cardiac surgery: evaluation of the RIFLE classification Ann Thorac Surg 2006 81 2 542 6 10.1016/j.athoracsur.2005.07.047 16427848

[B19] Bhat JG Gluck MC Lowenstein J Baldwin DS Renal failure after open heart surgery Ann Intern Med 1976 84 6 677 82 937879 10.7326/0003-4819-84-6-677

[B20] Slogoff S Reul GJ Keats AS Curry GR Crum ME Elmquist BA Role of perfusion pressure and flow in major organ dysfunction after cardiopulmonary bypass Ann Thorac Surg 1990 50 6 911 8 10.1016/0003-4975(90)91118-U 2241382

[B21] Witczak BJ Hartmann A Geiran OR Bugge JF Renal function after cardiopulmonary bypass surgery in patients with impaired renal function. A randomized study of the effect of nifedipine Eur J Anaesthesiol 2008 25 4 319 25 10.1017/S0265021507003158 18182121

[B22] Boldt J Brenner T Lehmann A Suttner SW Kumle B Isgro F Is kidney function altered by the duration of cardiopulmonary bypass? Ann Thorac Surg 2003 75 3 906 12 10.1016/S0003-4975(02)04559-9 12645715

[B23] Charlson ME MacKenzie CR Gold JP Ales KL Topkins M Shires GT Intraoperative blood pressure. What patterns identify patients at risk for postoperative complications? Ann Surg 1990 212 5 567 80 10.1097/00000658-199011000-00003 2241312 PMC1358184

[B24] Aronson S Fontes ML Miao Y Mangano DT Risk index for perioperative renal dysfunction/failure: critical dependence on pulse pressure hypertension Circulation 2007 115 6 733 42 10.1161/CIRCULATIONAHA.106.623538 17283267

[B25] Pirraglia PA Peterson JC Hartman GS Yao FS Thomas SJ Charlson ME The efficacy and safety of a pharmacologic protocol for maintaining coronary artery bypass patients at a higher mean arterial pressure during cardiopulmonary bypass J Extra Corpor Technol 1998 30 2 64 72 10182115

[B26] Valentine S Barrowcliffe M Peacock J A comparison of effects of fixed and tailored cardiopulmonary bypass flowrates on renal function Anaesth Intensive Care 1993 21 3 304 8 8342759 10.1177/0310057X9302100308

[B27] Urzua J Troncoso S Bugedo G Canessa R Munoz H Lema G Renal function and cardiopulmonary bypass: effect of perfusion pressure J Cardiothorac Vasc Anesth 1992 6 3 299 303 10.1016/1053-0770(92)90144-V 1610995

[B28] Gottesman RF Hillis AE Grega MA Borowicz LM Selnes OA Baumgartner WA Early postoperative cognitive dysfunction and blood pressure during coronary artery bypass graft operation Arch Neurol 2007 64 8 1111 4 10.1001/archneur.64.8.noc70028 17562924

[B29] Haines N Wang S Undar A Alkan T Akcevin A Clinical outcomes of pulsatile and non-pulsatile mode of perfusion J Extra Corpor Technol 2009 41 1 P26 9 19361037 PMC4680229

[B30] Cook DJ Proper JA Orszulak TA Daly RC Oliver WC Jr Effect of pump flow rate on cerebral blood flow during hypothermic cardiopulmonary bypass in adults J Cardiothorac Vasc Anesth 1997 11 4 415 9 10.1016/S1053-0770(97)90047-1 9187987

[B31] Kolkka R Hilberman M Neurologic dysfunction following cardiac operation with low-flow, low-pressure cardiopulmonary bypass J Thorac Cardiovasc Surg 1980 79 3 432 7 6965513

[B32] Kirklin JK Westaby S Blackstone EH Kirklin JW Chenoweth DE Pacifico AD Complement and the damaging effects of cardiopulmonary bypass J Thorac Cardiovasc Surg 1983 86 6 845 57 6606084

[B33] Murphy GS Hessel EA Groom RC Optimal perfusion during cardiopulmonary bypass: an evidence-based approach Anesth Analg 2009 108 5 1394 417 10.1213/ane.0b013e3181875e2e 19372313

