# Supplementary material for: Difference between pre-operative and cardiopulmonary bypass mean arterial pressure is independently associated with early cardiac surgery-associated acute kidney injury
Source: J Cardiothorac Surg. 2010 Sep 8;5:71. doi: 10.1186/1749-8090-5-71 (PMC2941753; doi:10.1186/1749-8090-5-71)
Supplement: Additional file 1 — Sensitivity multi-variable analysis exploring the association of delta MAP and CPB flow rate on post-operative CSA-AKI using an alternative definition for CSA-AKI. [file 1749-8090-5-71-S1.DOC]

**Additional file 1:** Sensitivity multi-variable analysis exploring the association of delta MAP and CPB flow rate on post-operative CSA-AKI using an alternative definition for CSA-AKI.

| Parameter | Odds Ratio | 95% CI | P-value |
| --- | --- | --- | --- |
| Male sex | 0.7 | 0.3-1.6 | 0.39 |
| Age ≥75 years (present) | 1.4 | 0.6-3.2 | 0.45 |
| BMI ≥25 kg/m^2^ (present) | 3.9 | 1.6-9.8 | 0.0035 |
| Relative delta MAP ≥26 mmHg (present) | 3.1 | 1.5-6.7 | 0.0034 |
| Flow ≥54 per mL/kg/min (present) | 0.3 | 0.09-0.5 | 0.0011 |
| Side-biting clamp (present) | 2.4 | 1.0-5.6 | 0.046 |

Abbreviations: BMI = Body Mass Index, MAP=Mean Arterial Pressure

C-Statistic = 0.78
